# Neuro-imaging characteristics of sensory impairment in cerebral palsy; a systematic review

**DOI:** 10.3389/fresc.2023.1084746

**Published:** 2023-03-17

**Authors:** A. C. S. Knijnenburg, C. V. M. Steinbusch, Y. J. M. Janssen-Potten, A. Defesche, R. J. Vermeulen

**Affiliations:** ^1^ Department of Neurology, Maastricht University Medical Centre+, Maastricht, Netherlands; ^2^ Research School MHeNS, Maastricht University, Maastricht, Netherlands; ^3^ Department of Rehabilitation Medicine, Adelante Rehabilitation Centre, Valkenburg, Netherlands; ^4^ Department of Rehabilitation Medicine, Maastricht University, Maastricht, Netherlands; ^5^ Research School CAPHRI, Maastricht University, Maastricht, Netherlands; ^6^ Department of Rehabilitation Medicine, Adelante Centre of Expertise in Rehabilitation and Audiology, Hoensbroek, Netherlands

**Keywords:** cerebral palsy, sensory function, functional connectivity, systematic review, somatosensory representation

## Abstract

**Background:**

*Objective:* To identify and examine neural reorganization of the sensory network in terms of lesion type, somatotopic organization of the primary somatosensory area, and functional connectivity in relation to sensory function in children and young adults with cerebral palsy (CP).

**Methods:**

*Design:* systematic review, Prospero registration ID 342570. *Data sources:* PubMed; Cochrane; Web of Science; Embase; CINAHL and PEDro from inception to March 13, 2021. *Eligibility criteria:* All types of original studies, concerning sensory connectivity in relation to sensory outcome in patients with spastic CP, <30 years of age. No publication status or date restrictions were applied. *Data extraction and synthesis:* Two authors independently determined the eligibility of studies. Quality assessment was performed by a third author. Neuro-imaging/neurophysiological techniques, sensory outcomes and patient characteristics were extracted.

**Results:**

Children and young adults with periventricular leucomalacia (PVL) lesions have significantly better hand function and sensation scores than patients with cortical-subcortical/middle cerebral artery (MCA) lesions. Ipsilesional reorganization of the S1 (primary somatosensory cortex) area appears to be the primary compensation mechanism after a unilateral early brain lesion, regardless of the timing of the lesion. Interhemispheric reorganization of the sensory system after early brain lesions is rare and, when it occurs, poorly effective. Diffusion tractography shows a positive correlation between the ascending sensory tract (AST) diffusivity metrics of the more affected hemisphere and sensory test outcomes.

**Discussion and conclusions:**

Because of the large variability in study design, patient characteristics, neuroimaging/neurophysiological techniques and parameters as well as sensory assessment methods used, it is difficult to draw definite inferences on the relationship between the reorganization of the sensory network following early brain damage and sensory function in children and young adults with CP. In general, sensory function seems to be worse in cortical as opposed to white matter tract (PVL) lesions. International consensus on a clinically relevant sensory test battery is needed to enhance understanding of the intriguing compensatory mechanisms of sensory network following early brain damage and potential consequences for rehabilitation strategies.

**Systematic Review Registration:**

https://www.crd.york.ac.uk/prospero/.

## Introduction

### Cerebral palsy

Cerebral palsy (CP) is a broad term for disorders of the development of movement and posture, causing activity limitation, which are attributed to non-progressive disturbances in the developing fetal or infant brain (1, 2). CP is one of the most common causes of physical disability in children, and the majority of these children have impaired hand function that makes them experience difficulties in performing daily activities (3, 4). It occurs in 2–3:1,000–1:2,500 live births (5, 6). Depending on the timing of the lesion during fetal development, different types of lesions occur; cortical maldevelopments (first and second trimester of gestation), periventricular white matter (PVL) lesions (early third trimester) or cortical and subcortical lesions/ middle cerebral artery infarctions (MCA) (around term age).

### Motor reorganization

The compensatory motor capabilities following early focal brain injury are intriguing, and these are reported to be superior to those of the adult brain. This observation, known as the Kennard principle, is based on a study of recovery following experimental lesions of the motor cortex in monkeys (7–9). Both lesion timing and cortical spinal tract (CST) wiring patterns have been shown to relate to upper limb function in children with unilateral CP (10). However, a large part of the variability in upper limb function still remains unexplained (7, 11).

### Sensory impairment

Aside from motor impairment, somatosensory impairment is also observed in children with CP. Somatosensory impairment is a broad term used for tactile deficits as well as for impairments in the processing of sensory information such as vibration, stereognosis, and two-point discrimination. In addition, proprioception can be considered one of the subsystems within the somatosensory system (12, 13). Proprioception consists of kinesthesia and joint position sense. Kinesthesia is the sense of extremity movement without visual input, and position sense is characterized by static limb position (14).

Thalamocortical projections start to reach the somatosensory cortex at the beginning of the third trimester. Conversely to CST wiring, developing thalamocortical somatosensory projections can still bypass even large periventricular brain lesions during this period (10). This tends to lead to sprouting to a broader area in the somatosensory cortex (15). Somatosensory processing is located in the primary somatosensory cortex (S1) in the postcentral gyrus of the parietal lobe and the secondary somatosensory cortex, located on the parietal operculum (16, 17). Median nerve stimulation, as well as object recognition, have been shown to activate the secondary somatosensory cortex (S2) bilaterally, regardless of the hand being stimulated, but only the contralateral S1 (18, 19). According to Auld et al., children with UCP performed worse in sensory tasks with their impaired hand compared to the unimpaired hand. However both hands performed worse than either hand of typically developing children. Over 75% of children with UCP have tactile deficits (20).

### Interaction motor and sensory system

Tactile deficits account for approximately 30% of the variance in upper-limb motor function in children with UCP (21). One example of this is the necessary sensory feedback in the modulation of fine motor tasks such as precision grip (15, 22). The majority of existing studies focus on especially motor performance, and there is only limited information about the involvement of the sensory system on functional outcome. However, understanding the extent and impact of sensory function on upper limb motor function is essential to improve rehabilitation approaches and functional outcomes (23).

### Aim of the study

In this systematic review we focus on all children and young adults with CP. However, experience shows that most studies on sensorimotor function concentrate on children and young adults with unilateral CP. Therefore, in the current study, we aimed to synthesize information on the consequences of brain damage (white matter characteristics, brain lesion types, functional connectivity) on somatosensory impairment and its impact on upper limb function in children and young adults with CP.

### Hypothesis

Based on the current literature, somatosensory impairment seems to be an important factor in upper limb dysfunction in children and young adults with CP (23). Therefore, we hypothesize a potential relation between neuroanatomical lesions, functional connectivity, and somatosensory impairment.

## Materials and methods

### Design

A systematic review was designed. The protocol has been registered in the National Institute for Health Research (NHS) on PROSPERO (International Prospective Register of Systematic Reviews) database: ID 342570.

### Data source and search strategy

A literature search was performed in six online databases: PubMed; Cochrane; Web of Science; Embase; CINAHL, and PEDro, from inception to March 13, 2021. Each search contained three main concepts: CP, MRI (magnetic resonance imaging), and sensation. The following Medical Subject Headings (MESH) terms and text words were used; ((((((Sensation) OR “Somatosensory Disorders”[Mesh]) OR “Sensation”[Mesh]) OR Sensory) OR Sensory function)) AND ((Cerebral palsy) OR Cerebral palsy [MeSH])) AND (((MRI) OR “Magnetic Resonance Imaging”[Mesh]) OR Magnetic Resonance Imaging). All results were uploaded to Rayyan Systems inc. (https://www.rayyan.ai/), an online tool to screen and select articles by multiple reviewers. Indicated duplicates were removed after checking by one of the first authors (CS). First, two review authors (CS, RV) independently screened titles and abstracts to remove irrelevant articles. Dissertation articles and conference abstracts were removed. Second, both authors reviewed the full text of potentially relevant studies to determine their eligibility. Consensus was reached on all articles.

### Inclusion criteria

The following inclusion criteria were applied: (1) human participants with spastic CP; (2) mean age of the participants was not older than thirty years of age, since the focus of the study included children and young adults; (3) MRI imaging available; (4) assessment of somatosensory function; (5) published in English, Dutch, French or German; (6) original research papers (exclusion of study protocols, reviews and conference abstracts).

### Data collection and data items

Our study objective was to investigate somatosensory deficits in relation to specific MRI abnormalities in children and young adults with spastic CP. The Preferred Reporting Items for Systematic Reviews and Meta-Analyses (PRISMA) guidelines were followed to extract the articles included in the review (24).

Data extracted included (1) first author and year of publication; (2) number and age of participants; (3) type of cerebral lesion; (4) clinical assessment of participants (motor and/or somatosensory assessment or categorization according to the Surveillance of Cerebral Palsy in Europe); (6) whether a control group was involved; (7) additional neuroimaging/neurophysiological method (e.g., electroencephalography, transcranial magnetic stimulation, somatosensory evoked potential, functional magnetic resonance imaging) (8) outcome of the studies.

### Quality assessment

Study quality was assessed (YJ) using the Standard quality assessment criteria for evaluating primary research papers from a variety of fields (25).

## Results

### General results

**Figure 1:**
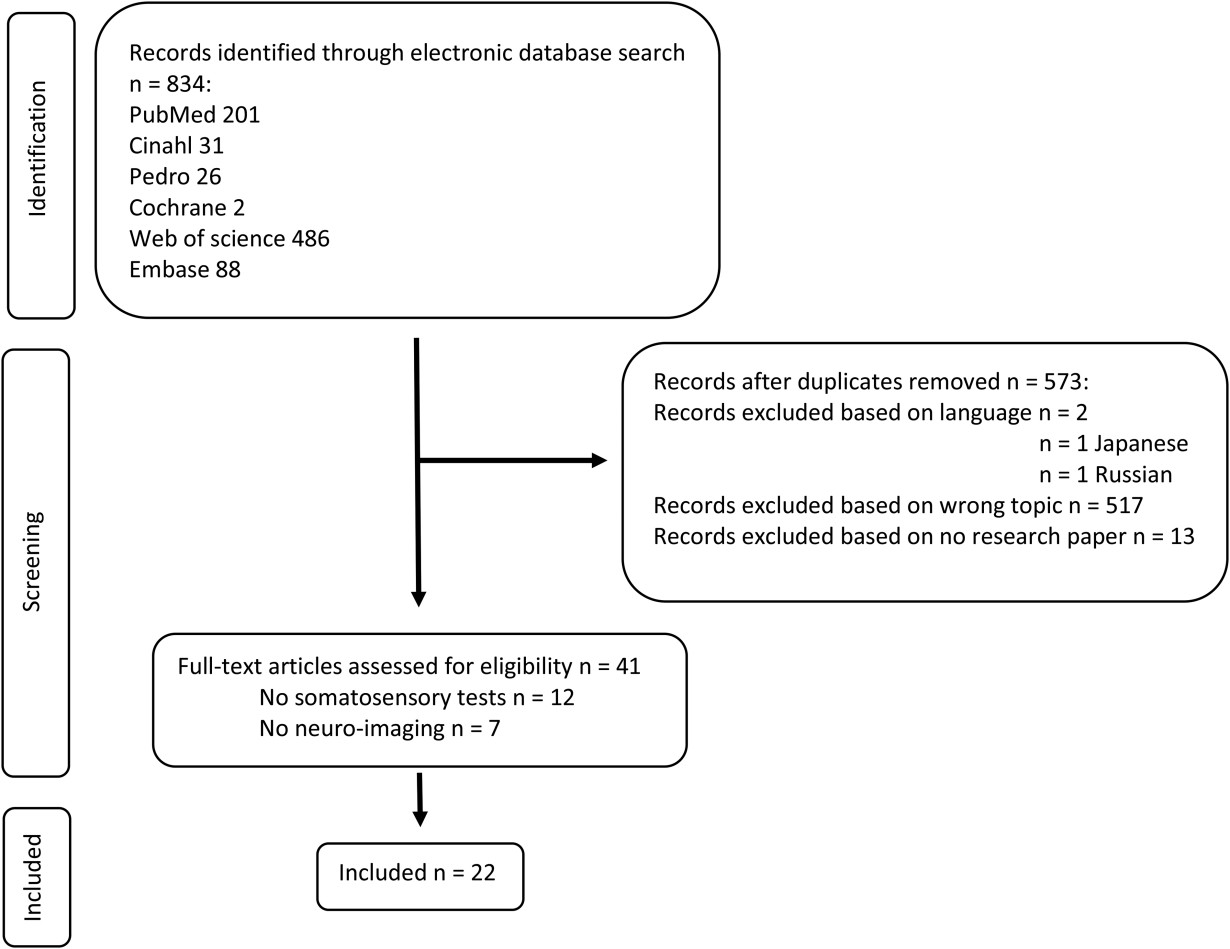
Flowchart of the study selection process.

After removing duplicates, the database search (last updated on March 13, 2021) yielded 573 citations. Twenty-two records were eventually included in the review. Figure 1 displays the flowchart of the study selection process according to the PRISMA guidelines (24).

The extracted information from the included articles is summarized in Table 1.

**Table 1:** Included studies in systematic review.

Study, year	Participants characteristics	Neuro-imaging	Other modalities	Neuro-imaging outcome measure	Functional sensory measures	Outcome
Dinomais, 2012 (26)	PVL *n* = 8 MCA *n* = 6 Age: 17 year (11–30 year)	MRI, fMRI	TMS	Functional connectivity in relation to grey matter damage	2PD	MCA group more sensory deficits and significantly reduced functional connectivity in the lesioned S2 (not in S1) compared with PVL group. When corrected for grey matter volume loss, no significant difference was found.
Feys, 2010 (7)	CMF *n* = 3 PVL *n* = 28 MCA *n* = 14 Acq. *n* = 8 Age: 9.9 year (5.3–14.2 year)	MRI		Lesion characteristics	Exteroception (touch), proprioception, 2PD, stereognosis	PVL: better performance. MCA and lesions basal ganglia/thalamus correlated to worse performance.
Fiori, 2015 (27)	PVL *n* = 50 GMFCS I–II MACS I–II Age: 11.4 year (5–17 year)	MRI, DTI diffusion semi-quantitative MRI score		Sq MRI scale vs. FA frontal, temporal, parietal, occipital	2PD, stereognosis	More severe lesions correlated with lower sensorimotor performance and impaired structural connectivity
Gupta, 2017 (28)	MCA *n* = 7 PVL *n* = 12 other *n* = 3 no MRI *n* = 2 Age: 10.5 year (±3.3 year)	MRI DTI	EEG, SSEP, TMS	Lesion characteristics, DTI ML, CST (SSEP, TMS CST wiring)	Stereognosis, 2PD	Both sensory and motor connectivity impact hand function in children with UCP. Disruptions in somatosensory connectivity and cortical lesions result in the most severe impairments
Guzzetta, 2007 (29)	MCA *n* = 3 PVL *n* = 3 CMF *n* = 6 Age: 16.8 year (10–28 year)	MRI, fMRI	SSEP TMS	Lesion characteristics, FMRI; perirolandic region and SMA (SSEP, TMS CST wiring)	tactile sense, pain and joint position sense, stereognosis, graphestesia and 2PD	Subjects with early brain lesion somato-sensory function generally reorganized within the affected hemisphere. A contralesional shifting is uncommon and poorly efficient in function restoration.
Hoon, 2009 (30)	PVL *n* = 28 21 spastic diplegia 4 quadriplegia 2 hemiplegia 1 ataxic/ hypotonic CP Age: 5 year 10 month (1,4–13y) Age matched controls TD *n* = 35	MRI, DTI		Lesion characteristics, DTI 26 tracts, posterior thalamic radiation, CST	Light touch, proprioception	Posterior thalamic radiation injury correlated with reduced contralateral touch threshold, proprioception, and motor severity, whereas corticospinal tract injury did not correlate with motor or sensory outcome measures.
Kuczynski, 2016 (31)	CSC *n* = 22 PVL *n* = 18 MACS I–II Age: 12 year (6–19 year) Age matched controls TD *n* = 60	MRI		Lesion type	Proprioception using KINARM Thumb and wrist position sense Thumb localization task, stereognosis, graphesthesia	Position sense impaired in MCA and PVL, remained when vision restored. Impairment is common and worse in arterial lesions
Kuczynski, 2017 (32)	PVL *n* = 20 CSC *n* = 23 MACS I–IV Age: 12 year (6–19 year) Age matched controls TD *n* = 60	MRI		Lesion type	Proprioception using KINARM Thumb and wrist position sense Thumb localization task, stereognosis, graphesthesia	Stroke cases displayed significantly impaired kinesthesia that remained when vision was restored. Kinesthesia was more impaired in arterial vs. venous lesions and correlated with clinical measures.
Kuczynski, 2017 (33)	CSC *n* = 14 PVL *n* = 15 Age: 12 year (6–19 year) Age matched controls TD *n* = 21	MRI, DTI		Lesion characteristics, mean FA, MD, RD, AD, and fiber count DCML	KINARM: Wrist and thumb position sense, thumb localization task. Stereognosis, graphesthesia	Sensory tract connectivity is altered in the affected hemisphere. Correlation between lesioned AST and proprioceptive measures.
Lemée, 2020 (34)	MCA *n* = 10 PVL *n* = 11 Age: 13.7 year (6 year 10 month–20 year 10 month)	MRI, fMRI		Lesion characteristics fMRI LI, S1, S2	2PD	High levels of contralesional activity associated with high levels of sensory impairment. Interhemispheric reorganization of the somatosensory system may not effectively compensate for somatosensory impairment
Mailleux, 2020 (35)	PVL *n* = 24 MCA *n* = 10 Age: 5–15 year	MRI, DTI	TMS	Lesion characteristics, DTI; CST, ML, TR and TC TMS (CST)	2PD, stereognosis	More damaged CST in MCA and ipsilesional CST projections. Correlations between diffusion metrics of targeted tracts and upper limb function. Asymmetry indices of the CST and sensory tracts could best explain bimanual performance and unimanual performance.
Papadelis, 2018 (15)	PVL *n* = 7 MCA *n* = 1 Other *n* = 2 GMFCS I (*n* = 9), GMFCS II (*n* = 1) MACS I (*n* = 5), MACS II (*n* = 5) Age: 12.3 year (±3.9 year) Age matched controls TD *n* = 13	MRI, DTI	MEG, with air pulses	Lesion characteristics, diffusion parameters AST, cortical mapping S1, Dig 1–5	Touch, 2PD	Bilateral changed cortical organization of S1 in HCP, associated with abnormalities in integrity of AST.
Perivier, 2016 (36)	PVL *n* = 11 MCA *n* = 10 BMFM 1–3 Age: 13 year 7 month	MRI		Grey matter volume S1, S2	2PD	Negative correlation between 2PD and gray matter volume ipsilesional S2 and S1, only significant in MCA.
Simon-Martinez, 2018 (37)	PVL *n* = 34 CSC *n* = 18 MACS I–III Age: 11 year 4 month (±3 year10 month)	MRI	TMS	Lesion characteristics CST wiring	Touch, 2PD, stereognosis, (movement, proprioception)	Sensory function predicted by lesion extent, timing and type of CST wiring.
Souza 2006 (38)	10 PVL *n* = 10 12 CSC *n* = 12 normal MRI *n* = 1 Age: 7–16 year Age matched controls TD *n* = 23	MRI		MRI lesion characteristics	sense of balance, sensing of vibration, touch, pain, temperature, distinguishing sharp objects, stereognosis, graphesthesia, 2PD; extinction.	Lesions in only one brain structure presented better results than those with two or more damaged structures larger than 10 mm. Patients with unilateral of bilateral cortical and subcortical impairment presented worse performance than those with subcortical lesions.
Thickbroom, 2001 (39)	PVL *n* = 7 Age: 25 year (15–57 year)	fRMI	TMS	fMRI active and passive movement	Proprioception stereognosis, graphesthesia, 2PD, touch, pain, vibration	Differences in reorganization of sensory and motor pathways in CP (motor contralesional, sensory ipsilesional).
Tsao, 2014 (40)	PVL *n* = 40 GMFCS I (*n* = 29), GMFCS II (*n* = 11) MACS I (*n* = 20), MACS II (*n* = 20) Age: 11.5 year (±3.1 year) Age matched controls TD *n* = 15	MRI. DTI		Diffusion parameters 34 cortical projections	2PD, stereognosis	Reduced connectivity were observed for connections with the primary motor cortex, primary sensory cortex and precuneus on the contralateral hemisphere in children with congenital hemiparesis.
Wilke, 2009 (41)	MCA (contra) *n* = 6 PVL (ipsi) *n* = 8 Age: 11–30 year	fMRI	MEG TMS	lesion characteristics, MEG identification S1, fMRI topographically variability, integrity somatosensory circuitry	Proprioception, 2PD, vibration	Normal pattern of somatosensory representation in both groups, no interhemispheric reorganization. Limited somatosensory compensatory potential.
Winckel, 2013 (42)	PVL *n* = 13 CSC *n* = 3 MACS I (*n* = 8), MACS II (*n* = 8) Age: 15 year (11–20 year) Age matched controls TD *n* = 18	fMRI		fMRI different brain areas during sensory discrimination task;	Extero, proprioception, 2PD, stereognosis	TD children more left frontal lobe and right cerebellum activation, CP children > left dorsal cingulate gyrus.
Winckel, 2013 (43)	PVL *n* = 14 CSC *n* = 3 MACS I *n* = 9, MACS II *n* = 8 Age: 14 year (11–19 year) Age matched controls TD *n* = 19	fMRI		fMRI different brain areas during active and passive movement and touch sensation	Exteroception (touch), proprioception, 2PD, stereognosis.	Ipsilateral cerebellar activity was seen in TD during all tasks and during active movements in CP. Additional ipsilateral S1 activation during passive movements and tactile stimulation.
Wingert, 2010 (44)	CP *N* = 10 GMFCS I (*n* = 6), GMFCS II (*n* = 4) MACS I (*n* = 5), MACS II (*n* = 5) Age: 18.6 year Age matched controls TD *n* = 10	fMRI		fMRI different brain areas during smooth, grated and shape discrimination	Touch, grating discrimination, proprioception	Reduced spatial extents in activated cortical areas and smaller BOLD response in cortical areas for somatosensation
Woodward, 2019 (45)	PVL *n* = 15 MACS I–IV Age: 9.1–14.5 year Age matched controls TD *n* = 21	fMRI + resting state connectivity		Resting state connectivity M1, S1, SMA, thalamus	Proprioception (KINARM)	Increased connectivity between non-lesioned S1 and thalamus/SMA = improved performance on proprioception.

Acq, acquired postnatally; AST, ascending sensory tract; BOLD, blood oxygen level dependent; CP, cerebral palsy; CST, corticospinal tract; CMF, cortical malformation; CSC, cortico-subcortical; DTI, Diffusion Tensor Imaging; fMRI, functional Magnetic Resonance Imaging; GMFCS, Gross Motor Function Classification Scale; KINARM, Kinesiological Instrument for Normal and Altered Reaching Movement; LI, lateralization index; MACS, Manual Ability Classification System; MEG, magnetoencephalography; MCA, middle cerebral artery; ML, Medial Lemniscus; MRI, Magnetic Resonance Imaging; PVL, periventricular leukomalacia; S1, somatosensory cortex; S2, secondary somatosensory cortex; SEP, Somatosensory Evoked Potentials; SMA, supplementary motor cortex; TD, typically developing; TMS, Transcranial Magnetic Stimulation; TC, sensorimotor transcallosal fibers; TR, superior thalamic radiations; y, year; 2PD, two point discrimination.

These twenty-two articles reported a total of 905 observations, 388 with a PVL-type lesion, 163 with an MCA-type lesion, 326 typically developing children (TD), and 25 with other lesions (Table 1). Most studies included children and young adults with a clinical unilateral cerebral palsy, except for the study of Wingert et al., which analyzed children with spastic diplegia, and Hoon et al., which analyzed children with mainly spastic diplegia (21/28) and also quadriplegia (4/28), hemiplegia (2/28) and ataxic CP (1/28) (30, 44). Next to MRI, five additional neuro-imaging/neurophysiological techniques were used. Sensory function was assessed in all studies. A combination of thirteen sensory assessments, i.e., tests, protocols as well as evaluation criteria, were used (see Table 1). For instance, stereognosis assessment protocols differed. Some studies used identification of six out of twelve familiar objects; three of six objects matched in pairs (pencil/pen, coin/button, paperclip/safety pin), and three of six differing objects (key, clothespin, marble, comb, spoon, ball) (7, 35, 42, 43). Whereas others used the number of correct responses out of a possible maximum of ten (28), nine (27, 40), five (29), six (37), or three objects(nickel, key, paperclip) (31–33). Two other studies did not include a detailed description of the stereognosis assessment (38, 39).

In almost all studies, motor function was reported, though not all studies reported on GMFCS or MACS. Some of the papers are from the same research groups (26, 31–33, 35, 37, 41–43, 45), potentially resulting in 312 overlapping observations. The results of the reports will be discussed according to sensory function.

Using the standard quality assessment criteria, most studies were of strong quality, with a total score above 0.80; one study was rated adequate and one good (25). The quality assessment is shown in Table 2.

**Table 2:** Quality assessment of included studies using kmet standard quality assessment criteria.

		Item 1	Item 2	Item 3	Item 4	Item 5	Item 6	Item 7	Item 8	Item 9	Item 10	Item 11	Item 12	Item 13	Item 14	Total sum	Total possible sum	Summary score
Dinomais et al. 2012	cohort study	2	2	1	2	N/A	N/A	N/A	2	1	2	2	N/A	2	2	18	20	0.90
Feys et al. 2010	cohort study	2	2	2	2	N/A	N/A	N/A	2	1	2	2	N/A	2	2	19	20	0.95
Fiori et al. 2014	cohort study	2	2	2	2	N/A	N/A	N/A	2	2	2	2	N/A	2	2	20	20	1.00
Gupta et al. 2017	cohort study	2	2	2	2	N/A	N/A	N/A	2	1	2	2	N/A	2	2	19	20	0.95
Guzetta et al. 2007	cohort study	2	2	2	2	N/A	N/A	N/A	2	1	2	2	N/A	2	2	19	20	0.95
Hoon et al. 2009	case-control	2	2	2	2	N/A	N/A	N/A	2	1	1	2	1	1	2	18	22	0.82
Kuczinski et al. 2016	case-control	2	2	2	2	N/A	N/A	N/A	2	2	2	2	2	2	2	22	22	1.00
Kuczinski et al. 2017	case-control	2	2	2	2	N/A	N/A	N/A	2	2	2	2	2	2	2	22	22	1.00
Kuczinski et al. 2. 2017	case-control	2	2	2	2	N/A	N/A	N/A	2	2	2	2	2	2	2	22	22	1.00
Lemee et al. 2019	cohort study	2	2	2	2	N/A	N/A	N/A	2	2	2	2	N/A	2	2	20	20	1.00
Mallieux et al. 2020	cohort study	2	2	2	2	N/A	N/A	N/A	2	1	2	2	N/A	1	2	18	20	0.90
Papedelis et al. 2018	case-control	2	2	1	2	N/A	N/A	N/A	2	1	1	2	1	2	1	17	22	0.77
Perivier et al. 2016	cohort study	2	2	1	2	N/A	N/A	N/A	2	2	2	2	N/A	2	2	19	20	0.95
Simon-Martinez et al. 2018	cohort study	2	2	2	2	N/A	N/A	N/A	2	2	2	2	N/A	2	2	20	20	1.00
Souza et al. 2006	case-control	1	1	1	1	N/A	N/A	N/A	2	1	1	2	1	1	1	13	22	0.59
Thickbroom et al. 2001	case reports	2	2	1	2	N/A	N/A	N/A	N/A	N/A	N/A	N/A	N/A	2	1	10	12	0.83
Tsao et al. 2014	cohort study	2	2	2	2	N/A	N/A	N/A	2	2	2	2	N/A	2	2	20	20	1.00
Wilke et al. 2009	cohort study	2	2	2	2	N/A	N/A	N/A	1	1	2	1	N/A	1	1	16	20	0.80
Winckel et al. 2013	case-control	2	2	2	2	N/A	N/A	N/A	2	2	2	2	2	2	2	22	22	1.00
Winckel et al. 2. 2013	case-control	2	2	2	2	N/A	N/A	N/A	2	1	2	1	2	2	1	19	22	0.86
Wingert et al. 2010	case-control	2	2	2	2	N/A	N/A	N/A	2	2	2	2	2	2	2	22	22	1.00
Woodward et al. 2017	case-control	2	2	2	2	N/A	N/A	N/A	2	2	2	2	2	2	2	22	22	1.00

### Tactile perception

#### Lesion characteristics

Patients with an MCA-type lesion have more severe deficits on two-point discrimination (2PD) test scores (7, 26, 28, 31, 32, 37, 41), stereognosis (7, 28, 31, 32, 37), and on graphesthesia test scores (31, 33), when compared with patients with a PVL-type lesion.

The location of the lesion is also correlated with performance on 2PD and stereognosis tests; Patients with basal ganglia and thalamic lesions (7, 37) have more severe deficits on 2PD and stereognosis tests. Patients with brainstem and temporal lobe lesions have more severe deficits on stereognosis tests. Patients with lesions in the corpus callosum and caudate have a more severe deficit on 2PD tests (27). In addition, larger lesions in these areas correlated with more severe deficits on 2PD and stereognosis tests (37, 38).

Patients with a disrupted CST and an ipsilateral wiring pattern, assessed by diffusion tensor imaging (DTI), have significantly more severe deficits in stereognosis tests (28, 35, 37). The relation between CST and performance on 2PD tests is less clear. Some studies found a correlation between damage and a more severe deficit on 2PD scores (35, 37), whereas others did not find this correlation (28).

#### Functional connectivity

In unilateral early brain lesions, an ipsilesional reorganization of the S1 area is found, regardless of the timing of the lesion (29, 34). Indeed, even in patients with brain malformations, contralateral shifting of the sensory function to the unaffected hemisphere is uncommon and poorly effective (29). Lemee et al. also showed most frequent contralateral pattern of sensory innervation, using brain activation with passive movement of the hand, suggesting intrahemispheric reorganization (34). Only in about 20% of the participants, an ipsilateral activation pattern was observed, coinciding with severe sensory deficits (34).

Within de S1 region, texture and shape recognition are impaired in damage to Brodmann area (BA)3, texture in BA1, and shape in BA2 (44).

S2 is predominately contralesional activated; however, high contralesional activation was associated with a more severe sensory deficit on 2PD tests (34). Patients with reduced functional connectivity in S2 (but not S1) had more severe sensory deficits on 2PD tests. However, when accounting for grey matter volume loss, this difference disappeared (26). Patients with gray matter volume loss in S1 and S2 had more severe sensory deficits on 2PD tests. This correlation was significant in patients with MCA-type lesions but not in PVL-type lesions (36).

Patients with either MCA-type lesions or PVL-type lesions, assessed with magnetoencephalography (MEG) and diffusion tensor imaging (DTI), showed larger distances between cortical representation areas of digits 1, 3, and 5 in the S1. In addition, in the more affected hemisphere, S1 was shifted anteriorly into the precentral gyrus. These increased distances in S1 digit representations are correlated with a more severe deficit on 2PD tests (15).

Patients with disrupted somatosensory connectivity of the ascending sensory tract (medial lemniscus), tested with Diffusion Tensor Imaging (DTI), have significantly more sensory deficit on 2PD and stereognosis tests as compared to those with intact somatosensory connectivity (15, 28, 33, 35, 40).

Patients with lower Fractional anisotropy of the medial lemniscus and higher mean diffusivity (MD) of the medial lemniscus, have more severe deficits on 2PD (15) and stereognosis tests (33, 35, 40). Patients with lower Fractional anisotropy of the superior thalamic radiations also have more severe deficits on 2PD and stereognosis. Low to no correlations were found between the diffusion metrics of the sensorimotor transcallosal fibers and sensory impairments (35). Patients with MCA-type lesions have more extensive differences in diffusion metrics compared to typically developing children. The differences in diffusion metrics between typically developing children and children with PVL-type lesions are limited (28, 33).

In patients with CP, an abnormal sensorimotor system, both anatomically (DTI) and electrophysiologically (EEG), was strongly correlated with abnormal motor function (Jebsen Taylor hand function test, Box and Blocks) and abnormal sensorimotor function (stereognosis and 2PD). The anatomy of the motor system, tested anatomically (DTI) correlated only weakly with bimanual function. Electrophysiology testing of the motor system (TMS) showed some correlation with the stereognosis (28). Using the lesion type classification, most effects could be explained by the more evident cortical involvement in the MCA-type lesions as compared to the PVL-type lesions.

Despite different motor and sensory reorganization patterns, the cortico-cerebellar circuitry, using functional MRI (fMRI), was well preserved in almost all patients and did not correlate with sensory deficits (26, 41, 43).

### Tactile registration

Tactile registration has been studied to a lesser extent. Hence the classification into “lesion characteristics” and “functional connectivity” has been discarded here. Feys et al. reported normal touch sensation in all children with PVL-type lesions, compared to approximately 75% of the children with cortical-subcortical brain lesions (7). Touch sensation could be significantly predicted by lesion timing, lesion location, and lesion extent (37, 38). In patients with PVL-type lesions, injury severity in thalamocortical pathways is related to sensation of touch (30, 37).

### Proprioception

#### Lesion characteristics

Lesion timing is not unambiguously associated to proprioception. In the study of Feys et al. no significant differences between lesion types were found (7), whereas Kuczynski et al. found position sense deficits to be more common and severe in children with MCA-type lesions (31, 32). Guzetta et al. included only one patient with an impaired position sense. This patient had the most severe sensory impairment (29).

Proprioception impairment is correlated with lesion location. Patients with posterior thalamic radiation injuries have more severe contralesional proprioception deficits (30).

Patients with lesions in only one brain structure have a better performance on proprioceptive tests compared to those with two or more damaged structures and lesions larger than 10 mm. Patients with unilateral or bilateral cortical and subcortical impairment have more sensory deficits (position sense, as well as tactile perception and registration tests) than patients with subcortical lesions (38).

#### Functional connectivity

Patients with MCA-type lesions and PVL-type lesions and lower FA, and higher MD, RD, and AD of the DCML tract, tested with DTI, have a more severe proprioceptive deficit (33). Patients with PVL-type lesions showed a more posteriorly and laterally organization of the AST compared with controls (33).

Patients with CP and decreased functional connectivity, tested with fMRI, between the non-lesioned S1 and thalamus/supplementary motor cortex (SMA) have a more severe position sense deficit. Whereas in typically developing children, position sense is positively correlated with connectivity between the thalamus and bilateral sensorimotor regions; increased connectivity is associated with poorer performance. Overall, the thalamus showed decreased connectivity in children with PVL-type lesions compared to controls (45).

In patients with CP, tested with fMRI, S1 activation is seen for active and passive movements as well as for tactile stimulation. There is additional ipsilateral S1 activation during passive movements and tactile stimulation. Ipsilateral cerebellar activity was observed in TD children during all tasks, but in CP children only during active movements (43). Typically developing children show more left frontal lobe and right cerebellum activation on fMRI during proprioceptive tasks compared to children with CP. Conversely, CP children activated the left dorsal cingulate gyrus to a greater extent than TD children (42).

One patient with a contralesional shift of primary sensory function, tested with SEP, was found; the responses in the ipsilateral hemisphere did not conform to latency and morphology of the response from the unaffected hand. This patient had the most severe position sense deficit (29).

## Discussion

### General

**Figure 2:**
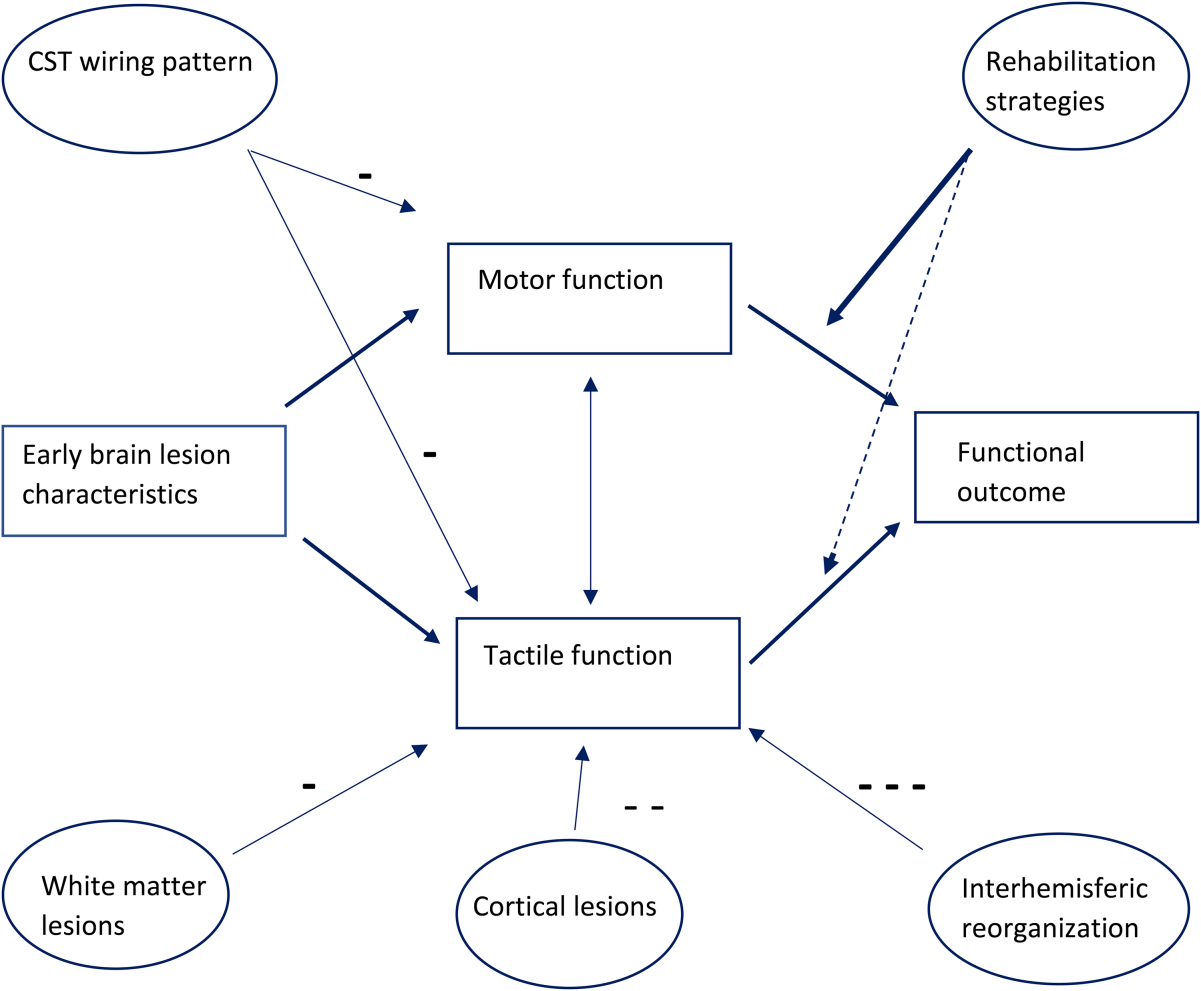
Diagram lesion relating to tactile, motor function and functional outcome, influencing anatomical lesions, rehabilitation strategies so far focused mostly on improvement of motor function.

Reorganization of the sensory system after early brain lesions is a complex and intriguing process. Although information on reorganization of sensory functions in children and young adults with CP is increasing, this reorganization process is still not fully understood. Understanding the pathophysiology of this reorganization process, and its relationship to sensory outcomes, and its relationship to its impact on functional outcomes might ultimately lead to different rehabilitation strategies, as shown schematically in Figure 2.

However, a comprehensive comparison of the evidence in the literature on sensory function in relation to anatomical lesions is difficult because different test batteries, sensory test protocols, and evaluation criteria are used, as well as different outcome measures for neuro-imaging, as shown in Table 1. Moreover, when the available sensory information is associated with lesion timing, lesion location, lesion extent, and functional connectivity, studies tend to focus on one particular tract or lesion type. In addition, we need to take into account that currently used protocols for sensory assessment most likely underestimate the sensory deficits in patients with CP (22). Therefore, international consensus on comprehensive sensory test batteries, protocols, and evaluating criteria is necessary to allow comparison of somatosensory function in relation to brain injury characteristics and ultimately influence functional outcome with personalized rehabilitation strategies.

Most of the literature on sensorimotor function in CP focuses on children and young adults with unilateral CP. The results of our search confirmed this; while the intent was to include all children with CP, most studies included patients with clinical unilateral cerebral palsy and mild impairments. The study of Wingert et al., and Hoon et al., included a large/solely group of participants with spastic diplegia. The results of these studies were included in this systematic review because they were in our intended patient category. Even in the unilateral cerebral palsy groups, especially in case of white matter damage, the abnormalities were often bilateral, meaning even though the patients have a clinical unilateral CP the groups are more similar in lesion type than clinically expected. Further, the results of the study of Hoon et al. are in line with the study of Mailleux et al. and the study of Wingert et al. uses a different study protocol (grating discrimination), making the risk of bias low (30, 35, 44).

There is substantial evidence that patients with PVL-type lesions have significantly less sensory deficits as opposed to patients with cortical-subcortical/MCA-type lesions (7, 27, 28, 31, 32, 34, 37, 38, 41). Lesion extent, type of CST wiring pattern, and lesion location significantly further impact sensory function (7, 37).

In the next paragraphs, we will elaborate on the relationship between specific neuroanatomical abnormalities and specific sensory deficits and the potential consequence for rehabilitation in more detail.

### Tactile perception

Damage to cortical and subcortical structures reduces the likelihood of the CST trajectory developing in the typical contralateral pathway. In case of ipsilateral CST wiring, the association between sensory and motor functions is disrupted. This points toward a different mechanism of sensorimotor integration in patients with CP as a probable cause for the association between CST wiring and increased sensory deficits (39). The relation between CST wiring and stereognosis is found across multiple studies (28, 35, 37). Some studies found a correlation between CST wiring and poorer performance of the 2PD test, while others did not find this correlation (35, 37). However, there was a large spread of 2PD test scores in this group, which possibly explains the lack of a statistical significant difference (28).

Ipsilesional reorganization of the S1 area appears to be the primary compensation mechanism after a unilateral early brain lesion, regardless of the timing of the lesion. Developing thalamocortical somatosensory projections can still bypass even large periventricular brain lesions during the third trimester (10). This tends to lead to sprouting to a broader area in the somatosensory cortex, and the wider distances correlate with impaired sensory function (15). Somatosensory deficits in patients with PVL-type lesions are better explained by loss of integrity of these thalamocortical pathways than by loss of grey matter volume in ipsilesional S1 and/or S2. The loss of grey matter volume in S1 and S2 in these patients was not related to sensory outcome (36). In patients with MCA-type lesions and subsequent volume loss of the grey matter in S1 and S2, the reorganization capabilities of these thalamocortical pathways are less, resulting in a more severe deficit. The observed inter-hemispheric reorganization of S2 (34) could be related to the observed bilateral activation of S2 after tactile stimulation, seen in healthy volunteers (16, 19). However, high contralesional activation was associated with a severe impairment of sensory function, making this compensatory mechanism inadequate to say the least (34).

Diffusion tractography shows a positive correlation between the ascending sensory tract (AST) axial diffusivity (AD) of the more affected hemisphere and sensory test outcomes. Increased axial diffusivity may indicate gliosis and structural abnormalities in the integrity of the AST (15, 33, 40). These differences are more extensive in patients with MCA-type lesions compared to patients with PVL-type lesions, implying that damage to the ascending sensory tracts is more extensive in patients with MCA-type lesions (33). This is consistent with the finding that patients with MCA-type lesions have more severe sensory deficits. Posterior thalamic radiation injury also correlates with sensory impairment (30, 35).

Cortico-cerebellar circuits, measured using functional MRI (fMRI), were well preserved in almost all patients. Despite this well-preserved cortico-cerebellar circuitry, no correlations were found with sensory deficits. Thus, this intact circuitry did not compensate for sensory deficits (26, 41, 43).

### Proprioception

When assessing proprioceptive deficits in relation to lesion types, this relationship is less clear. In the study of Feys et al. no significant differences in proprioception between lesion types were found (7). In contrast, Kuczynski et al. found deficits in position sense to be more common and also more severe in children with MCA-type lesions (31, 32). Guzetta et al. included only one patient with an impaired position sense; this patient had the most severe sensory impairment (29). It should be noted that in all studies a substantial number of patients with normal proprioception were included, which potentially caused a bias in the conclusions (7, 29, 31–33, 42, 43). Another potential explanation lies in the assessment itself, since sensory deficits are most likely to be underestimated in patients with CP (22).

Functional connectivity between the non-lesioned S1 and thalamus/SMA in patients with cerebral palsy is inversely correlated to position sense; higher functional connectivity is associated with better performance. Whereas in typically developing children, position sense is positively correlated with connectivity between the thalamus and bilateral sensorimotor regions; increased connectivity is associated with poorer performance. Overall, the thalamus showed decreased connectivity in children with PVL-type lesions compared to controls, suggesting that early lesions can disrupt sensory network components, and connectivity between these areas is related to tactile perception deficits (45).

There is limited evidence of interhemispheric reorganization of the somatosensory functions, only two studies reported on patients with an interhemispheric reorganization, and these patients had the most severe sensory deficit. So when an interhemispheric reorganization is observed, this reorganization does not lead to improvement of sensory function (29, 34).

### Neurorehabilitation and sensory deficits

In recent years, rehabilitation programs have paid more attention to enhancing sensory functions during CIMT/HABIT(ILE) programs (23, 46–49) and in study protocols (50–52). As in our systematic review, these studies show a large variability in study design, patient characteristics, and sensory assessment methods used. This makes it difficult to compare the findings. No distinction was made based on lesion type across these studies/ in the study protocols. However, all studies found an improvement in one or more sensory domains, making it worthwhile to explore these differences and the effect of lesion characteristics on the ability to achieve these differences (23, 46–49). In a study on adult stroke patients, different altered patterns of cortical activation were observed following touch discrimination training when patients with thalamic/capsular lesions were compared with patients with S1/S2 cortical somatosensory lesions. These changes were different despite common training and similar improvement (53). If the ability to change cortical activation in relation to lesion type and in relation to sensory improvement could be unraveled, a foundation could be laid for a more individualized training program.

### Study limitations

There are several limitations to be considered. Due to the large variability in study design, patient characteristics, neuroimaging/neurophysiological techniques and outcome parameters, and sensory assessment methods used, only a partial synthesis of evidence was possible. In addition, some of the papers included in this systematic review used the same study population. Ten of the twenty-two articles included described at least one patient over eighteen. Not wanting to exclude a large portion of the studies and thus missing essential observations, led to the selection of papers including both children and young adults in this systematic review. The populations are by nature small and often heterogeneous. Several papers included children with relatively minor sensory deficits, which potentially caused a bias in the conclusions (29, 42, 43).

When reviewing the literature, another paper was discovered, which should have been included in the original search. This paper by Chu et al. researched the reorganization of hand somatosensory cortex in children with CP (54). Fortunately, we did not miss any essential information because this study shows similar results to the papers included in the original search. Key words in the paper of Chu were “*perinatal brain injury*” as opposed to “*cerebral palsy*” used in our original search terms. This might be the reason this paper was not included in the original search.

As we included only original research papers, the reviews of Brun et al. (12) and Poitras et al. (13), in which somatosensory deficits in children with cerebral palsy were discussed, were not selected for this review. Although neural correlates were mentioned, the main focus in these reviews was on different sensory domains. The review of Garberova et al. focused on somatosensory function in relation to fMRI, (functional magnetic resonance imaging), disregarding other modalities (55). This makes our review complementary to these reviews.

### Conclusion and recommendation

In conclusion, it is hard to draw definite inferences on the relationship between the reorganization of the sensory network following early brain damage and sensory function in children with CP because of the large variability in study design, patient characteristics, neuroimaging/neurophysiological techniques, and parameters and sensory assessment methods used. In general, lesion timing, lesion location, lesion extent, integrity of the ascending sensory tract, and structural abnormality of the somatosensory areas have an impact on sensory function. In line with these observations, patients with cortical MCA lesions have more severe sensory deficits across all sensory modalities as opposed to patients with white matter (PVL) lesions. Intrahemisferic reorganization is the most common type of reorganization of the sensory system. In addition, an interhemisferic reorganization of the sensory system was associated with poor sensory function. International consensus on a clinically relevant sensory test battery is needed to enhance understanding of the intriguing compensatory mechanisms of sensory network following early brain damage and potential consequences for rehabilitation approaches.

## Data Availability

The original contributions presented in the study are included in the article/Supplementary Material, further inquiries can be directed to the corresponding author.
